# Studying relationships between environment and malaria incidence in Camopi (French Guiana) through the objective selection of buffer-based landscape characterisations

**DOI:** 10.1186/1476-072X-10-65

**Published:** 2011-12-13

**Authors:** Aurélia Stefani, Emmanuel Roux, Jean-Marie Fotsing, Bernard Carme

**Affiliations:** 1 EPat Team (EA 3593), UFR de Médecine - Université des Antilles et de la Guyane, Cayenne, French Guiana; 2 ESPACE-DEV, UMR 228 IRD/UMII/UR/UAG, Institut de Recherche pour le Développement, Cayenne, French Guiana; 3 Laboratoire Hospitalo-Universitaire de Parasitologie Mycologie, Cayenne General Hospital, French Guiana; 4 Centre d'Investigation Clinique - Epidémiologie Clinique Antilles Guyane (CIE 802 INSERM), Cayenne General Hospital, French Guiana

**Keywords:** Malaria, *Plasmodium falciparum*, *Plasmodium vivax*, environmental risk factors, landscape modelling, remote sensing, buffer, model selection, Akaike information criterion.

## Abstract

**Background:**

Malaria remains a major health problem in French Guiana, with a mean of 3800 cases each year. A previous study in Camopi, an Amerindian village on the Oyapock River, highlighted the major contribution of environmental features to the incidence of malaria attacks. We propose a method for the objective selection of the best multivariate peridomestic landscape characterisation that maximises the chances of identifying relationships between environmental features and malaria incidence, statistically significant and meaningful from an epidemiological point of view.

**Methods:**

A land-cover map, the hydrological network and the geolocalised inhabited houses were used to characterise the peridomestic landscape in eleven discoid buffers with radii of 50, 100, 200, 300, 400, 500, 600, 700, 800, 900 and 1000 metres. Buffer-based landscape characterisations were first compared in terms of their capacity to discriminate between sites within the geographic space and of their effective multidimensionality in variable space. The Akaike information criterion (AIC) was then used to select the landscape model best explaining the incidences of *P. vivax *and *P. falciparum *malaria. Finally, we calculated Pearson correlation coefficients for the relationships between environmental variables and malaria incidence, by species, for the more relevant buffers.

**Results:**

The optimal buffers for environmental characterisation had radii of 100 m around houses for *P. vivax *and 400 m around houses for *P. falciparum*. The incidence of *P. falciparum *malaria seemed to be more strongly linked to environmental features than that of *P. vivax *malaria, within these buffers. The incidence of *P. falciparum *malaria in children was strongly correlated with proportions of bare soil (r = -0.69), land under high vegetation (r = 0.68) and primary forest (r = 0.54), landscape division (r = 0.48) and the number of inhabited houses (r = -0.60). The incidence of *P. vivax *malaria was associated only with landscape division (r = 0.49).

**Conclusions:**

The proposed methodology provides a simple and general framework for objective characterisation of the landscape to account for field observations. The use of this method enabled us to identify different optimal observation horizons around houses, depending on the *Plasmodium *species considered, and to demonstrate significant correlations between environmental features and the incidence of malaria.

## Background

Malaria is a major public health problem in French Guiana, a French overseas region located in South America. This territory, separated from Brazil and Suriname by the Oyapock and Maroni rivers, respectively, is largely covered by the Amazon forest (occupying 94% of its area). About 3800 acute cases of malaria are recorded in French Guiana each year. Most transmission occurs inland, along the rivers, whereas the coastal areas inhabited by 75% of the population are almost free of transmission [1]. An epidemiological study in Camopi, an Amerindian village at the eastern border of French Guiana, reported a global incidence of 935 per 1000 person-years for children under the age of five years, with 70% of cases caused by *P. vivax *and the remaining 30% caused by *P. falciparum*. This investigation highlighted the predominant role played by environmental factors in the areas surrounding households: clearing of vegetation, and distances to the river and the forest [2].

*Anopheles darlingi*, an efficient vector species common in the Americas, is widely distributed in French Guiana and has been considered the primary vector of malaria for 50 years in this territory [3,4]. This species has highly anthropophilic behaviour and its activity in the coastal area of French Guiana has a bimodal rhythm, with a peak at dusk and another at dawn, superimposed over nocturnal activity peaking in the middle of the night [5]. Anopheles darlingi is the major anopheline species collected in Camopi [6], but its role in transmission at this site has yet to be clearly demonstrated. The reported maximum flight range of *An. darlingi *is 7 km [7], but the distance covered depends heavily on conditions such as the presence of humans, landscape features and climate parameters. The precise conditions in which vector-borne transmission occurs, potentially accounting for malaria endemicity and epidemic events in French Guiana, particularly in Camopi, therefore remain unclear [6,8]. Improvements in our understanding of the impact of environmental factors on malaria incidence would provide us with better insight into these mechanisms.

Remote sensing (RS) and geographic information systems (GIS) have emerged as methods for exploring environmental factors potentially associated with vector-borne diseases in health studies [9]. Indeed, RS has often been used in epidemiological studies of parasitic diseases (59% of such studies), including malaria (16% of studies) [10]. The analysis of spatial patterns in entomological data by RS and GIS methods has been used in the mapping of potential vector breeding sites and the identification of areas at risk of malaria [11-24]. Satellite imagery has also been used to model the spatial risk of malaria, based on the relationship between land-cover and climatic features on the one hand, and the prevalence or incidence of malaria on the other [25-35].

These latter approaches are consistent when epidemiological and environmental data are aggregated in space and time in order to result in the spatial coincidence of geo-localised cases and associated transmission locations and the temporal coincidence of these cases with periods of transmission. However, in the absence of firm hypotheses concerning the locations at which transmission occurs, studies of vector bioecology, with the finer spatial and temporal resolution of data, are required if we are to understand the mechanisms of disease transmission [36-38].

Furthermore, in studies involving spatial components, it is essential to determine the most appropriate spatial scale for the biological process considered. By examining buffers of several sizes around reference sites at which the studied phenomenon is measured, it is possible to determine the most appropriate observation horizon [24,39].

In this study, we explored the relevance of a local-scale study of relationships between landscape characterisation and georeferenced malaria cases. Associations between landscape features, characterised by remote sensing, and the peridomestic risk of disease have already been investigated for Lyme disease, at the scale of individual properties [40,41]. Very few studies investigating landscape characterisation at this very local scale and its relationship to the incidence of malaria have been published [42]. With very little information available concerning the spatial and temporal distribution of the disease vector, work at this scale necessitates the formulation of firm hypotheses concerning the likely sites of transmission. Thus, in this study, we hypothesised that most transmission occurs in and around dwellings. We therefore assumed that georeferenced malaria cases could be considered to characterise exposure to mosquito vectors and thus to identify the location and time of contamination, at least for young children (before the acquisition of immunity and autonomy in movements). We begin by proposing an objective method for selecting the best buffer-based landscape characterisations (in terms of landscape composition and structure) from a set of candidates differing in the radius of the discoid buffer used. We then calculated Pearson correlation coefficients between malaria incidence data and environmental variables for the selected buffers. Finally, we discuss our results from the standpoints of both methodology and application.

## Methods

### Study area

The study was conducted in Camopi, a village on the Oyapock River, which serves as the border between French Guiana and Brazil. This village consists of a main central hamlet and 28 hamlets within a 15 km^2 ^area, on the banks of the Oyapock and Camopi Rivers. The 1200 registered inhabitants in 2009 were mostly Amerindians of the Wayampi and Emerillon ethnic groups, living on the banks of the Oyapock and Camopi Rivers, respectively. These groups have a traditional lifestyle, practising subsistence slash and burn agriculture [43], fishing, hunting and gathering. These Amerindian populations, which were formerly nomadic, have become sedentary following the implantation of public services, such as a health centre and elementary and secondary schools, and improvements in their living conditions have resulted in very high rates of population growth. In this context, the area under crops is gradually increasing. The people live in wood huts, known locally as "carbets", which have a roof of palm leaves, steel sheeting or tarpaulin. Nevertheless, modern concrete houses are progressively replacing these traditional dwellings, particularly in the principal hamlet. Camopi is isolated from the inhabited coastal region and the nearest town, Saint-Georges de l'Oyapock, is located at 100 km downstream on the Oyapock River. Tourism is not permitted in Camopi, and special authorisation is required for all non-residents wishing to reach the village.

### Cohort of children

We carried out an open cohort study of children under the age of seven years, followed from January 1^st^, 2001 to December 31^st^, 2009. Included individuals were all the children born between January 1^st^, 1994 and December 31^st^, 2008. Malaria data were not censored after the first malaria infection, and children were followed up until they reached the age of seven years. Data for children for whom follow-up was interrupted were right censored at the date of interruption. We assumed that all bouts of malaria were recorded at the local health centre, given the isolation of the population, its limited mobility and the almost systematic frequentation of the health centre in cases of fever [44].

### Clinical and parasitological data

A bout of malaria (or acute clinical episode of malaria) was defined as fever (temperature ≥ 38°C at the time of consultation or during the previous 48 hours) associated with a thin blood smear positive for asexual forms of *Plasmodium*. Blood smears were initially examined in Camopi by nurses trained in microscopy and were subsequently checked at the Laboratory of Parasitology and Mycology of Cayenne Hospital. When blood smear examinations were not feasible, rapid diagnostic tests (OptiMAL^® ^test) were performed. A list of all acute clinical malaria episodes, the date of diagnosis, the *Plasmodium *species present and parasitaemia (when available) was established. Relapses of *Plasmodium vivax *infection in French Guiana have a purely tropical pattern, with a short latent period (Chesson strain) [45]. Therefore, for a given child, each *P. vivax *malaria episode occurring within 90 days of the previous *P. vivax *malaria episode was considered to be a relapse and was therefore withdrawn from the statistical analysis and the calculation of incidence rate for transmission [46]. Thanks to this filtering of the data, we can reliably assume that the annual incidence of bouts of *P. vivax *malaria corresponds to the annual incidence of transmission. This approach makes the investigation of relationships between the environment and *P. vivax *malaria incidence consistent. The observation of differences in the temporal patterns of *P. vivax *and *P. falciparum *incidence rates suggests that there may be two different transmission mechanisms at work, involving different environmental conditions and, possibly, vectors [47]. We therefore also analysed the incidence rates of *P. vivax *and *P. falciparum *malaria separately. Incidence data were finally aggregated at the level of the hamlet, as incidence rates at household level would be subject to large errors due to the small number of children per *carbet*.

### Ethical considerations

The protocol was approved by the information processing in research in the field of health committee (CCTIRS) and the national data protection agency (CNIL). Informed consent was provided by one of the parents of each child before inclusion in the study. Written consent forms were signed by the investigator, the parent and the interpreter before completion of the questionnaire. All diagnosed bouts of malaria were treated.

### Land-cover and landscape characterisation

#### Land-cover characterisation

**Figure 1:**
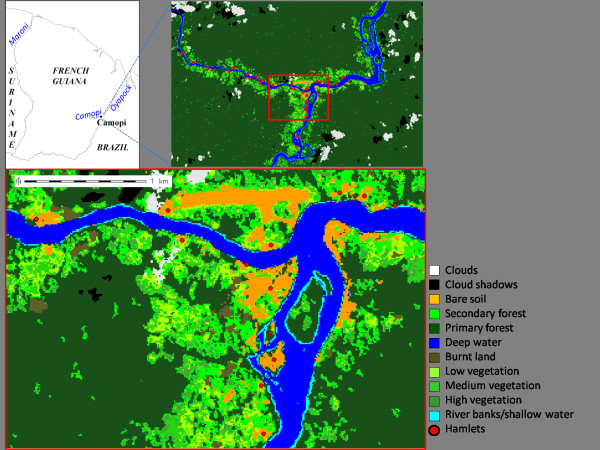
**Land-cover characterisation of the study site, with a magnification of the confluence of the Oypapock and Camopi Rivers**.

Land-cover characterisation was based on a colour SPOT 5 Satellite image acquired during the dry season, on August 30^th^, 2006. The image used has a spatial resolution of 10 m and a four-band spectral resolution. Semi-supervised classification was performed with GRASS GIS 6 software, to characterise land-cover. Photographs taken from the air, with a spatial resolution of 50 cm, acquired by the French National Geographic Institute in 2006 (BD-ORTHO^® ^product), were interpreted by eye, for the labelling of classes identified on the satellite image and for qualitative validation of the classification. In total, nine classes were identified: *primary *and *secondary forest*, *high*, *medium *and *low vegetation*, *body of water*, *burned area*, *bare soil *and *river banks/shallow water *(see Figure 1). *Unfragmented forest *was defined as the unbroken patch (*i.e. *the set of adjacent pixels belonging to the same class) of primary forest surrounding the village. A land-cover map of this type was also used in a previous study by Girod *et al. *[6].

#### Complementary environmental/geographic data

Data on rivers and creeks not visible on SPOT 5 satellite images due to their small size and/or the dense vegetation cover were extracted from the BD CARTHAGE^® ^product of the French National Geographic Institute (IGN). BD CARTHAGE^® ^is the hydrographic reference system for France, produced in 2009 for French Guiana by the Regional Direction of the Environment (DIREN) of Guiana and the French National Agency for Water and Aquatic Environments (ONEMA). For the area studied, this database was generated from the digital elevation model provided by the Shuttle Radar Topography Mission (SRTM), with a spatial resolution of 30 metres.

All households were geolocalised with a global positioning system (GPS) -- Magellan^® ^eXplorist™ 600 -- or by digitalisation from the aerial photographs of BD-ORTHO^®^.

#### Landscape characterisation (modelling)

For each household, the surrounding landscape was characterised within discoid buffers of different radius (50 m, 100 m and then every 100 m until 1000 m) in terms of the percentage of each land-cover category, the length of the Camopi and/or Oyapock river banks, the length of creeks, the number of inhabited houses and two measurements of landscape division [48]: one taking all land-cover classes other than *deep water *into account (referred to as *landscape division 1 *below) and the other taking into account all land-cover classes other than *deep water *and *unfragmented forest *(referred to as *landscape division 2 *below). This second measurement of landscape division was designed to take into account only anthropised areas in the calculation of landscape division. As mentioned by Jeager [48], "the degree of landscape division is defined as the probability that two randomly chosen places in the landscape under investigation are not situated in the same undissected area".

In fact, taking into account deep water bodies in the computation led to a systematic underestimation of the landscape fragmentation and a less discriminant characterisation. The second measurement of landscape division was designed to take into account only anthropised areas in the calculation of landscape division.

According to Jeager [48], the two landscape division computations are obtained with the following formula:

$$Landscape\;division{\;}_{k}=\text{1}-\underset{i\in I_{k}}{\sum }{\left(\frac{A_{i}}{\underset{i\in I_{k}}{\sum }A_{i}}\right)}^{\text{2}}$$

where *A_i _*is the area of the *i^th ^*patch, *p_i_*, of land cover/use, *k *∈{1,2}, *I_1 _= *{*i */*p_i _∉ Deep water*} and *I_2 _= *{*i/p_i _∉ Deep water AND p_i _∉ unfragmented forest*}.

Households presenting more than 20% missing data (presence of clouds and/or cloud shadows) were excluded.

Landscape features at the household level were averaged at the level of the hamlet for investigations of their relationship to incidence. So, for each of the 28 hamlets of the village of Camopi, eleven (corresponding to the number of buffer sizes) landscape characterisations were generated, each one including 14 environmental variables.

### Selecting the best landscape characterisation

We used two complementary approaches, based on different criteria, for objective selection of the best landscape characterisation from the 11 candidates. The first considered only environmental data and was designed to ensure i) significant discrimination of hamlets within the geographic space and ii) non information redundancy within the environmental variable space; the second also made use of epidemiological data and selected the landscape characterisation best explaining the incidence of malaria within a multiple linear regression framework.

In the common situations where environment is characterised by a multivariate data set and where very few background knowledge are available on data and processes involved, the selection of a consistent and informational landscape characterisation should satisfy the previous criteria.

#### Data preprocessing

Variables that clearly had a highly asymmetric distribution were subjected to square-root transformation before processing. Square-root transformation clearly provided better overall results than logarithmic transformation. Variables were transformed for all buffers, to make it possible to compare results for different buffer sizes and to facilitate interpretation. The variables transformed in this manner were: *P. falciparum malaria incidence*; *number of inhabited dwelling*; *length of river banks*; *length of creeks*; *and the proportions of land under bare soil*, *burnt*, with *medium vegetation *and *river banks/shallow water*.

#### Evaluation of the informational content of the landscape characterisations

**Figure 2:**
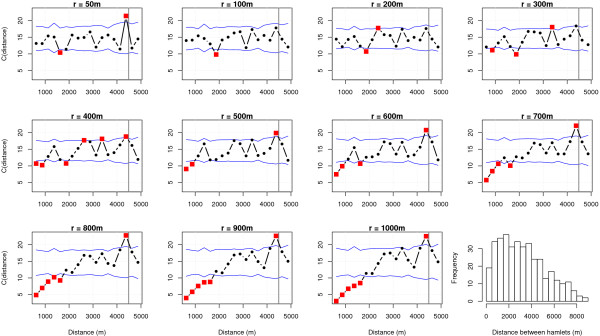
**Multivariate variograms of environmental variables as a function of buffer size**. The envelope corresponds to the 95^th ^and 5^th ^quantiles of the distribution of 10000 variograms obtained after random permutations of the environmental data. Squares represent significant values, *i.e. *values below the 5^th ^quantiles or above the 95^th ^percentile. The vertical line corresponds to the distance beyond which the variogram is not interpretable, *i.e. *half the maximum distance between villages. The last graph shows a histogram of hamlet distances.

Intuitively, as the inter-buffer overlap increases with the buffer sizes, the larger the buffers are the less discriminant they are for hamlets that are close from one to another. So we first determined the spatial variance of the buffer-based environmental variables, with multivariate variograms [49,50] computed for 18 distance classes of 250 m in width, from 500 to 5000 m. Variogram significance was assessed by computing 10000 variograms after random permutations of the environmental data, making it possible to calculate the 5^th ^and the 95^th ^percentiles for each distance class (see Figure 2).

From a complementary point of view, we noticed an increase of the inter-correlation level of the environmental variables when the buffer sizes increased. In other terms, large buffers led to a significant information redundancy among the variable set, i.e. a loss of the effective multi-dimensionality. So, the overall correlation between environmental variables was then evaluated simply by calculating the mean absolute Pearson correlation coefficient for all pairs of variables. This coefficient provides information about the richness and complementarity of the information contained in the variables. This coefficient increases with the redundancy of the information.

#### Data-driven model selection

Evaluations of the informational content of the different landscape characterisations based on environmental data alone do not, by definition, take into account the data we are trying to explain: malaria incidence in our case. We therefore selected the candidate characterisation best explaining the observed pattern of incidence in the framework of a multiple linear regression. This approach relates to the problem of model selection. We suggested the use of a model selection procedure based on that described by Dray *et al. *[49] and Roux *et al. *[51]. For each buffer size, a principal component analysis (PCA) was performed on environmental variables. Principal components (PCs) were then normed and sorted in descending order, according to their capacity to account for the response variable (malaria incidence, by species), *i.e. *according to the proportion of the variance accounted for by a linear regression model. The sorted PCs (considered as explanatory variables) were entered one by one into a multiple regression model. Principal components are linearly uncorrelated. This ensures that the multiple linear regression is not performed on collinear explanatory variables and gives stable results. A new model was thus defined for each new entry of an explanatory variable. This procedure resulted in the construction of the same number of models as there were PCs. The corrected Akaike information criterion (AICc) was then calculated for each model. AICc is a corrected version of AIC for small sample sizes (28 hamlets here), making it possible to select the best model in terms of two antagonistic criteria: accuracy and parsimony. The best model was considered to be that giving the lowest AICc value.

### Correlations between the incidence of malaria and land-cover characteristics

Pearson correlation coefficients were calculated for the relationships between malaria incidence, by species, and the variables associated with the selected environment characterisation. Correlation coefficients between malaria incidence rates and buffer-based environmental variables were computed only for the buffers selected by means of the previous analysis.

## Results

### Epidemiological results

A total of 541 children were included into this cohort. Transmission incidence rates were 248, 292 and 11 per 1000 person-years for *P. falciparum, P. vivax *and mixed infections, respectively. We considered 43% of the bouts of *P. vivax *malaria initially included to correspond to relapses, which were therefore removed from the analysis. A detailed description of the epidemiological results is presented in Stefani *et al. *[47].

### Missing data due to cloud cover

One of the 29 hamlets constituting the village of Camopi was entirely under cloud, precluding the characterisation of land-cover. For another hamlet, five of the six *carbets *presented more than 20% missing data for the 50 m and 100 m-radius buffers and were excluded from the study.

### Discrimination power in geographic space - variograms of environmental data

We generated multivariate variograms [50] of the environmental variables as a function of buffer size, and a histogram of the distances between all possible pairs of hamlets (Figure 2). Only buffers of 200 to 400 m gave significantly high variances for hamlets separated by 2500 to 3500 m, providing a significant discrimination in geographic space for these distances. Twenty-one percent of all the hamlet pairs exhibit distances comprised within this interval. So a non negligible part of the set of hamlets can be spatially discriminated by choosing buffer radii between 200 to 400 m.

On the contrary, larger buffer radii, especially above 600 m, are associated with higher levels of spatial autocorrelation for hamlets located in close proximity. In particular, for radii above 800 m, hamlets distant of less than 1750 m from one to another are significantly correlated from the environmental point of view. Yet, 40.0% of all the possible hamlet pairs exhibit a distance lower than 1750 m and thus a significant spatial auto-correlation.

### Evaluation of the dimensionality within the environmental variable space

**Figure 3:**
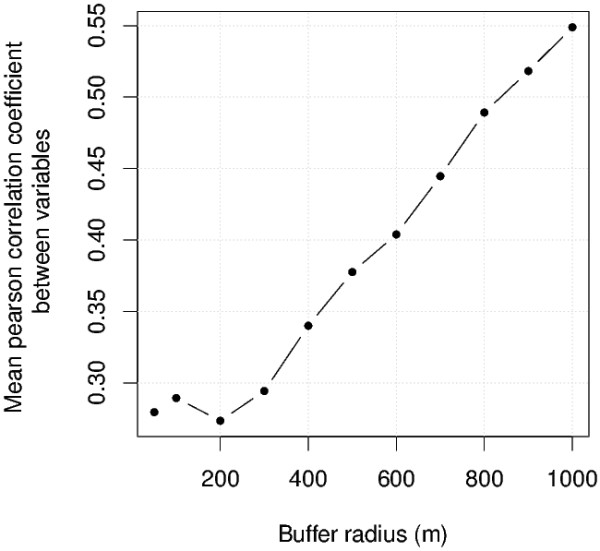
**Mean absolute values of Pearson's correlation coefficients for all pairs of variables**.

Figure 3 shows the mean absolute values of Pearson correlation coefficients for all pairs of environmental variables.

For buffers with a radius above 400 m, the correlation between the variables increased strongly with buffer size, reducing the richness of the information contained in the variables and, thus, their potential explanatory power. The minimum of the information redundancy is reached for a buffer of 200 m.

As a preliminary conclusion, by considering the two last environmental variable investigations, we state that multivariate, discriminant and informative landscape characterisations are provided by buffers with radii of at most 400 m. However, the following model selection procedure was applied to larger buffers (up to 600 m) in order to discuss the consistency of this procedure results with the previous ones.

### Selection of the model best explaining malaria incidence

**Figure 4:**
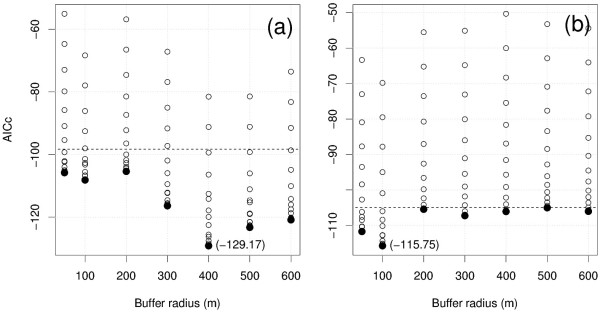
**AICc values as a function of the buffer sizes and for (a) *P. falciparum *and (b) *P. vivax***. Filled circles correspond to the minimum values and numbers in brackets correspond to the AICc values for the best models. The horizontal dashed lines correspond to the AICc values for the "null" models (i.e. with only intercepts) above which the models are not valid.

Figure 4 presents the results for the AICc value distributions as a function of the buffer sizes.

**Figure 5:**
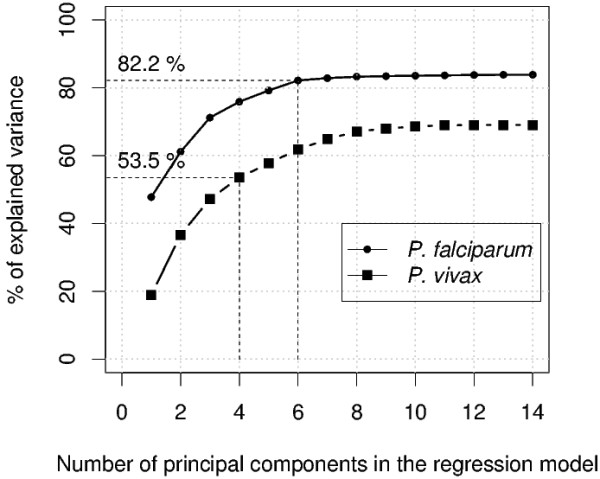
**Variance accounted for by the multiple regression models obtained with buffers of 100 m (*P. vivax*) and 400 m (*P. falciparum*)**.

The best compromise between model accuracy and parsimony was obtained with a buffer of 100 m for the incidence of *P. vivax *malaria (r^2 ^= 0.54) and for a buffer of 400 m for the incidence of *P. falciparum *malaria (r^2 ^= 0.82). These two models had four and six components, respectively (see Figure 5). The six components of the selected *P. falciparum *model were PCs numbers 1, 14, 10, 3, 2 and 4, in decreasing order of explanatory capacity. For *P. vivax*, the selected regression model included PCs numbers 11, 2, 4 and 14. Moran's index of spatial autocorrelation was calculated for the residuals of the two models and for a large range of weighted neighbourhood structures. No correlation was found in either case, the p-value being exceeding 0.63 for *P. falciparum *and 0.06 for *P. vivax *model residuals, regardless of the weighted neighbourhood structure.

The four primary axes of the PCA for environmental variables, accounting for 81.8% of the total variance of the environmental variables, appeared in the *P. falciparum *regression model (the six PCs defining the regression model accounted for 82.2% of the total environmental variable variance). In particular, the first PCA principal component, accounting for 36.7% of the total variance of the environmental data, was the first variable selected in the construction of the regression model. This was not the case for the best *P. vivax *regression model. The four PCs included in the best regression model for *P. vivax *accounted for only 35.3% of the total variance of the environmental variables. In particular, the first PC selected (the 11th) was not informative or discriminating from an environmental viewpoint.

### Interpretation of principal components

An exploration of the contribution of variables to the definition of the factorial axis, for the *P. vivax *model, showed that PC 11 could be considered a "dense vegetation" variable (the variables contributing most to this axis, together making up 80% were *medium vegetation, secondary forest, landscape division, primary forest and bare soil*). PC 2 could be considered an "anthropogenic" variable (*bare soil, number of inhabited houses, burnt area, deep water, length of river banks and river banks/shallow water*). PC 4 could be considered a "river banks and vegetation" variable (*river banks/shallow water, medium vegetation, secondary forest, primary forest and length of river banks*) and PC 14 was a mixture of all types of environmental variables (*bare soil, primary forest, deep water and secondary forest*). In the *P. falciparum *model, PC 1 could be considered a "dense vegetation, anthropogenic and water body" variable (*high vegetation, number of inhabited houses, bare soil, length of creeks, primary forest, length of river banks and deep water*). PC 14 could be considered a "forest" variable (*primary forest, deep water and bare soil*). PC 10 could be considered a "dense vegetation and anthropogenic" variable (*medium vegetation, number of inhabited houses, secondary forest, length of river banks, landscape division 2 and deep water*). PC 3 could be considered a mixture of all types of variables (*secondary forest, river banks/shallow water, landscape division 2, bare soil and primary forest*). PC 2 could be considered a "low vegetation" variable (*low vegetation, medium vegetation, landscape division 2 and landscape division*) and PC 4 a "fragmentation and high vegetation" variable (*burnt area, landscape division, high vegetation, secondary forest, landscape division 2, river banks/shallow water and length of river banks*).

### Correlation between incidence and initial environmental data

Pearson correlation analyses of the relationships between environmental variables and the incidences of *P. vivax *and *P. falciparum *within buffers of 100 and 400 m, respectively, are presented in Table 1. Five variables were significantly (p < 0.01) associated with *P. falciparum *incidence: proportions of bare soil (r = -0.69), high vegetation (r = 0.68) and primary forest (r = 0.54), landscape division (r = 0.48) and number of inhabited dwellings (r = -0.60); whereas *P. vivax *incidence was associated only with landscape division (r = 0.49).

**Table 1:** Linear correlations between initial environmental variables and *P. vivax *and *P. falciparum *incidences.

	*P. vivax*		*P. falciparum#*	
**Variable**	**Pearson r**	**P value**	**Pearson r**	**P value**

% bare soil#	-0.17	0.381	**-0.69**	< 0.001**
% secondary forest	0,00	0.999	-0.03	0.891
% primary forest	0.08	0.688	**0.54**	0.003**
% deep water	0.28	0.149	**-0.39**	0.043*
% burned land#	-0.24	0.218	**-0.43**	0.022*
% low vegetation	-0.09	0.665	0.06	0.761
% medium vegetation#	-0.05	0.783	0.24	0.227
% high vegetation	0.17	0.396	**0.68**	< 0.001**
% river banks/shallow water#	0.30	0.124	-0.24	0.212
No. of inhabited dwellings#	-0.30	0.116	**-0.60**	0.001**
Length of river banks#	0.32	0.097	**-0.44**	0.018*
Length of creeks#	-0.06	0.771	**0.48**	0.01*
Landscape divison 1	**0.46**	0.013*	-0.10	0.601
Landscape divison 2	**0.49**	0.008**	**0.48**	0.009**

We used buffers with radii of 100 m and 400 m for *P. vivax *and *P. falciparum *incidences, respectively. Symbols * and ** correspond to statistical significance at the 0.05 and 0.01 (alpha risks) levels, respectively. Significant correlation coefficients are shown in bold. Landscape division 2 was computed by considering all land-cover classes other than *unfragmented forest*. The symbol # identifies variables that have been transformed (square-root transformation).

## Discussion

We first chose to characterise the environment around each household, and then averaged such characterisation for each hamlet. Direct landscape characterisation around hamlets could appear more straightforward but presented two main drawbacks: firstly it demands to clearly materialise the hamlet entity as a geographic object, which is not easy and would result of arbitrary choices (point corresponding to the barycentre of hamlet dwellings, or surface or line corresponding to the convex hull of the dwellings, etc.); secondly, the environment surrounding the hamlet can differ from the one surrounding the individual dwellings and, among them, the dwellings which are inhabited by children included in the cohort.

From a methodological standpoint, we propose here a simple and general framework for an objective and informative multivariate landscape characterisation and when very few background knowledge are available on involved processes. This is a frequent problem in landscape ecology and solutions are often determined arbitrarily, particularly when there is no available ethological knowledge (*e.g. *dispersion capacity) to guide an objective choice. In this context, we initially proposed tools that consider environmental variable features only: the multivariate variogram evaluating the capacity of the landscape characterisation to discriminate sites in the geographic space; the mean absolute Pearson correlation coefficient for pairs of environmental variables, which provides an indication of the redundancy within the environmental variable space. However, the most original part of the data analysis methodology relates to the objective selection of the best landscape characterisation, by means of a data-driven model selection procedure based on multiple linear regression and the Akaike information criterion.

The proposed method is not restricted to the case of discoid buffers or to studies of malaria incidence. For instance, it could be applied to the parameterisation of IFM-like (incidence function model-like) measurements, as described in Moilanen and Nieminen [52]. A comparable application of the proposed methodology was described by Roux et al. [51] for selection of the most appropriate spatial weighted structure for modelling the presence and abundance of the insect vector of Chagas disease.

The methodology provides results that may be sensitive to many factors other than the outcome variable (in this case, malaria incidence). The most important of these factors is the set of environmental variables used for landscape characterisation. Moreover, the environmental data preprocessing may affect the results. In our case, a logarithmic transformation was applied, and the results were compared with those of square-root transformation. No significant difference was found in model structures, but the results, in terms of model accuracy and Pearson correlation coefficients, were poorer.

In the context of our application, the first steps of the proposed methodology tended to eliminate large buffers (radius > 400 m), which gave poor spatial discrimination of hamlets and displayed high levels of information redundancy for environmental variables. The data-driven selection model was then used to identify the optimal observation horizons: 100 and 400 m buffers were found to be the most appropriate for characterising the environment when considering *P. vivax *and *P. falciparum *malaria incidences, respectively.

The four primary PCs of the PCA for environmental variables were included in the *P. falciparum *regression model selected. The hamlets were thus similarly structured (or ordered) both environmentally and epidemiologically. There was therefore a strong link between environmental features and the incidence attributed to this malaria species. By contrast, the association between *P. vivax *malaria incidence and environmental characteristics seemed to be weaker.

In Pearson's linear correlation analysis, the proportion of bare soil within the 400 m buffer zone was found to be associated with protection against *P. falciparum *malaria. This land-cover feature was not favourable for the rest of adult mosquitoes or for the maintenance of breeding sites. It was closely linked to the density of dwellings, which was also found to be predictive of the incidence of *P. falciparum *malaria. Children living in isolated houses therefore had an increased risk of *P. falciparum *malaria. The proportions of primary forest and high vegetation were correlated with a higher incidence of *P. falciparum *malaria. This finding is consistent with previously published results [18,19,53]. According to Tadei and coworkers, *An. darlingi *returns to the forest after feeding when houses are located close to forest [54-57]. The composition of the high vegetation class requires confirmation in the field, but includes plants, shrubs and relatively small trees, contrasting with the composition of primary forest, at the interface of crop areas and secondary or primary forest. It may correspond to the vegetation present at least five years after deforestation described by Olson et al. [58]. Moreover, the length of creeks was positively correlated with *P. falciparum *incidence, whereas the length of river banks was negatively correlated with this incidence. Thus, vector breeding sites are probably located mostly along small streams (creeks) rather than along the banks of the main river. Moreover, deep water appeared to be a factor protective against malaria, probably because it provides neither suitable breeding sites nor resting sites for adult mosquito vectors. This counterbalances the contribution of short distance to the main river as a risk factor for transmission [2] and justifies further investigations of the role of river banks in the development of breeding sites for Anopheles.

The percentage of burnt land was negatively correlated to malaria incidence. However, this land use is very transitory in space and time, replacing primary forest, secondary forest or high vegetation and preceding soils with poor vegetation cover and low vegetation over a period of a few months. Traditionally, Amerindians in Camopi burn their crop lands from the middle to the end of the dry season (*i.e. *from the end of August to the end of November). At the time at which the image was taken, the burnt lands were linked to villages with a low malaria incidence. We therefore suspect that there may be confounding factors linked to spatial distribution, as burning activity did not occur at the same time at all the hamlets. It is therefore not possible to determine the real effect of burning.

Landscape division within 100 and 400 m buffers was associated with higher incidences of *P. vivax *and *P. falciparum *malaria, respectively. Greater fragmentation of the landscape was therefore associated with a higher incidence of malaria, suggesting that anthropogenic presence and activity, which tend to increase landscape fragmentation and ecological changes, probably increase malaria incidence by favouring the presence and development of malaria [57,59-61].

Classification was processed from an image taken in the dry season. Due to the topography of the study area and particularly of the river banks, the water level of the main rivers (Camopi and Oyapock) does not influence river bank positions to an extent that could be characterised by the 10-metre spatial resolution optic images (except during extreme and not representative events). However, some rocks appear in the rivers during low water level periods. They could increase the bare soil proportion in some buffers but to a negligible proportion. On the other hand, Camopi is located in humid tropical forest and the dense and almost permanent cloud cover in rainy season simply prevents us to obtain exploitable optic images during this period. In such a context, high resolution radar images could provide useful information.

The links between clinical bouts of malaria and the periods and sites of contamination are simpler and more direct in young children. Indeed, this population has little specific immunity (especially younger children) and their exposure is limited to their dwelling or to the village, depending on their age. Furthermore, in this study, malaria data were collected by following up an exhaustive cohort in a "captive" general population (*i.e. *all the children are followed from birth, with diagnosis occurring at only one place, and the access to diagnosis sources being unlimited). However, although the environment directly accounts for the abundance of the vector and, thus, the sites and extent of transmission, it cannot entirely account for the clinical data registered at the health centre, even for the children in Camopi. Several biases must be taken into account, such as i) individual genetic susceptibility to malaria and its clinical expression; ii) the protective measures used (nets, repellents, etc.) and iii) whether consultation at the health centre was systematic for the diagnosis of all episodes of fever (self-medication, traditional treatment, etc.). A difference in genetic susceptibility between the two ethnic groups has been reported [2]. More than 75% of the children of the cohort spend all their nights under mosquito nets and more than 70% of the families use insecticides or topical repellents (personal results). Finally, we assumed that all bouts of malaria were recorded at the local health centre, due to the isolation of the population and its limited mobility [44]. Moreover, with the chosen rule for identifying *P. vivax *relapses [46], some false-negative and false-positive new *P. vivax *infections may remain in the database. A bias in the exclusion of relapses and, thus, in the quality of *P. vivax *data might account for the weak link between environmental data and this malaria incidence for this species.

The behavioural habits of the families such as the use of bed nets or insecticides could have introduced a bias into the analysis. Nevertheless, in a multivariate Cox modelling approach [47], these variables were not risk factors for malaria attacks in children. Consequently, in the present study, we decided not to take into account these parameters.

*Anopheles darlingi *has not been implicated with certainty in the bouts of malaria occurring in Camopi, but this study nonetheless focused on young children, based on a hypothesis of nightly transmission at home, due to the characteristics of *An. darlingi *[3]. However, other studies have reported *An. darlingi *to be active 24 hours per day and to be found outside during the day [5], suggesting that some transmission may occur in places frequented by children during the day. Furthermore, other anopheline species may be involved in malaria transmission, including during the morning [4,62]. The age composition of the *An. darlingi *population may depend on season and environment [63,64]. Thus, the involvement of another anopheline species or of different populations of *An. darlingi *in *P. vivax *transmission than in *P. falciparum *transmission may account for the weak relationship between the environment and *P. vivax *incidence at the peridomestic scale of observation.

There are limitations to the usefulness of RS for epidemiology [10], but this tool has several advantages: it objectively characterises the landscape features associated with malaria incidence and makes it possible to assess the sensitivity of the results to buffer size. It also provides access to past information and can be used for mapping and spatial analysis, which are useful for control measures. Furthermore, RS may provide additional information not collected by field surveys, in which observations are limited to short distances.

Our results suggest that the use of buffers of 100 and 400 m around houses is the most appropriate, in this specific case, for demonstrating a characteristic land-cover pattern accounting for differences in incidence rates as a function of the species concerned. This is greater than the radius of observation that can be covered by the human eye in the field. On the basis of this modelling, it is possible to establish a predictive map of *P. falciparum *malaria risk in Camopi. However, for this to be achieved correctly, we must consider not only buffer-based landscape features, but also factors such as distance to each land-cover class, and non-environmental data, such as the socio-economic and behavioural characteristics of the local populations.

## Conclusions

The application of a simple and general model selection method led to the identification of different optimal observation horizons around dwellings as a function of the *Plasmodium *species involved. This study also shows that, assuming -- based on the cohort composition -- domestic or peridomestic transmission, very significant relationships between environmental data and malaria incidence can be highlighted at a very local scale. These results suggest that it may be possible to develop an environment-based predictive model of the incidence of malaria in this neotropical rainforest area.

## Competing interests

The authors declare that they have no competing interests.

## Authors' contributions

AS participated in research design, data collection, analysis and interpretation, and prepared the manuscript. ER participated in research design, data analysis and interpretation and revised the manuscript. JMF was involved in data interpretation and revised the manuscript. BC designed the cohort and was involved in data interpretation and manuscript revision. All authors read and approved the final manuscript.

## References

[B1] Carme B Ardillon V Girod R Grenier C Joubert M Djossou F Ravachol F [Update on the epidemiology of malaria in French Guiana] (in French) Med Trop 2009 69 19 25 19499726

[B2] Hustache S Nacher M Djossou F Carme B Malaria risk factors in Amerindian children in French Guiana Am J Trop Med Hyg 2007 76 619 625 17426159

[B3] Floch H La lutte antipaludique en Guyane française. L'anophélisme Riv Malariol 1955 24 57 65 13255704

[B4] Mouchet J Nadire-Galliot M Poman JP Claustre J Bellony S Le paludisme en Guyane. Les caractéristiques des différents foyers et la lutte antipaludique Bull Soc Pathol Exot 1989 82 393 405 2670293

[B5] Pajot F-X Le Pont F Molez J-F Degallier N Agressivité d'Anopheles (Nyssorhuynchus) darlingi Root, 1926 (Diptera, Culicidae) en Guyane française Cah ORSTOM, sér Ent méd et Parasitol 1977 15 15 22

[B6] Girod R Roux E Berger F Stefani A Gaborit P Carinci R Issaly J Carme B Dusfour I Unravelling the relationships between Anopheles darlingi (Diptera: Culicidae) densities, environmental factors and malaria incidence: understanding the variable patterns of malarial transmission in French Guiana (South America) Ann Trop Med Parasitol 2011 105 107 122 10.1179/136485911X12899838683322 21396247 PMC4084657

[B7] Charlwood JD Alecrim WA Capture-recapture studies with the South American malaria vector Anopheles darlingi, Root Ann Trop Med Parasitol 1989 83 569 576 2619371 10.1080/00034983.1989.11812389

[B8] Basurko C Hanf M Han-Sze R Rogier S Héritier P Grenier C Joubert M Nacher M Carme B Influence of climate and river level on the incidence of malaria in Cacao, French Guiana Malar J 2011 10 26 10.1186/1475-2875-10-26 21294884 PMC3042423

[B9] Beck LR Lobitz BM Wood BL Remote sensing and human health: new sensors and new opportunities Emerging Infect Dis 2000 6 217 227 10.3201/eid0603.000301 10827111 PMC2640871

[B10] Herbreteau V Salem G Souris M Hugot J-P Gonzalez J-P Thirty years of use and improvement of remote sensing, applied to epidemiology: from early promises to lasting frustration Health Place 2007 13 400 403 10.1016/j.healthplace.2006.03.003 16735137

[B11] Beck LR Rodriguez MH Dister SW Rodriguez AD Rejmankova E Ulloa A Meza RA Roberts DR Paris JF Spanner MA Remote sensing as a landscape epidemiologic tool to identify villages at high risk for malaria transmission Am J Trop Med Hyg 1994 51 271 280 7943544 10.4269/ajtmh.1994.51.271

[B12] Beck LR Rodriguez MH Dister SW Rodriguez AD Washino RK Roberts DR Spanner MA Assessment of a remote sensing-based model for predicting malaria transmission risk in villages of Chiapas, Mexico Am J Trop Med Hyg 1997 56 99 106 9063370 10.4269/ajtmh.1997.56.99

[B13] Achee NL Grieco JP Masuoka P Andre RG Roberts DR Thomas J Briceno I King R Rejmankova E Use of remote sensing and geographic information systems to predict locations of Anopheles darlingi-positive breeding sites within the Sibun River in Belize, Central America J Med Entomol 2006 43 382 392 10.1603/0022-2585(2006)043[0382:UORSAG]2.0.CO;2 16619625

[B14] Pope KO Rejmankova E Savage HM Arredondo-Jimenez JI Rodriguez MH Roberts DR Remote sensing of tropical wetlands for malaria control in Chiapas, Mexico Ecol Appl 1994 4 81 90 10.2307/1942117 11539870

[B15] Rakotomanana F Jeanne I Duchemin JB Pietra V Raharimalala L Tombo ML Ariey F Approche géographique dans la lutte contre le paludisme dans la région des Hautes Terres Centrales à Madagascar Archives de l'Institut Pasteur de Madagascar 2001 67 27 30 12471743

[B16] Machault V Vignolles C Pagès F Gadiaga L Gaye A Sokhna C Trape J-F Lacaux J-P Rogier C Spatial heterogeneity and temporal evolution of malaria transmission risk in Dakar, Senegal, according to remotely sensed environmental data Malar J 2010 9 252 10.1186/1475-2875-9-252 20815867 PMC2944340

[B17] Rodriguez AD Rodriguez MH Hernandez JE Dister SW Beck LR Rejmankova E Roberts DR Landscape surrounding human settlements and Anopheles albimanus (Diptera: Culicidae) abundance in Southern Chiapas, Mexico J Med Entomol 1996 33 39 48 8906903 10.1093/jmedent/33.1.39

[B18] Zhou G Munga S Minakawa N Githeko AK Yan G Spatial relationship between adult malaria vector abundance and environmental factors in western Kenya highlands Am J Trop Med Hyg 2007 77 29 35 17620627

[B19] Minakawa N Munga S Atieli F Mushinzimana E Zhou G Githeko AK Yan G Spatial distribution of anopheline larval habitats in Western Kenyan highlands: effects of land cover types and topography Am J Trop Med Hyg 2005 73 157 165 16014851

[B20] Sainz-Elipe S Latorre JM Escosa R Masià M Fuentes MV Mas-Coma S Bargues MD Malaria resurgence risk in southern Europe: climate assessment in an historically endemic area of rice fields at the Mediterranean shore of Spain Malar J 2010 9 221 10.1186/1475-2875-9-221 20673367 PMC2924348

[B21] Tran A Ponçon N Toty C Linard C Guis H Ferré J-B Lo Seen D Roger F de la Rocque S Fontenille D Baldet T Using remote sensing to map larval and adult populations of Anopheles hyrcanus (Diptera: Culicidae) a potential malaria vector in Southern France Int J Health Geogr 2008 7 9 10.1186/1476-072X-7-9 18302749 PMC2291038

[B22] Munga S Yakob L Mushinzimana E Zhou G Ouna T Minakawa N Githeko A Yan G Land use and land cover changes and spatiotemporal dynamics of anopheline larval habitats during a four-year period in a highland community of Africa Am J Trop Med Hyg 2009 81 1079 1084 10.4269/ajtmh.2009.09-0156 19996440 PMC3726196

[B23] Mushinzimana E Munga S Minakawa N Li L Feng C-C Bian L Kitron U Schmidt C Beck L Zhou G Githeko AK Yan G Landscape determinants and remote sensing of anopheline mosquito larval habitats in the western Kenya highlands Malar J 2006 5 13 10.1186/1475-2875-5-13 16480523 PMC1420309

[B24] Diuk-Wasser MA Toure MB Dolo G Bagayoko M Sogoba N Sissoko I Traore SF Taylor CE Effect of rice cultivation patterns on malaria vector abundance in rice-growing villages in Mali Am J Trop Med Hyg 2007 76 869 874 17488907 PMC1945821

[B25] Hernández-Avila JE Rodríguez MH Betanzos-Reyes AF Danis-Lozano R Méndez-Galván JF Velázquez-Monroy OJ Tapia-Conyer R Determinant factors for malaria transmission on the coast of Oaxaca State, the main residual transmission focus in Mexico Salud pública Méx 2006 48 405 417 17063824 10.1590/s0036-36342006000500007

[B26] Brooker S Leslie T Kolaczinski K Mohsen E Mehboob N Saleheen S Khudonazarov J Freeman T Clements A Rowland M Kolaczinski J Spatial epidemiology of Plasmodium vivax, Afghanistan Emerg Infect Dis 2006 12 1600 1602 10.3201/eid1210.060051 17176583 PMC1773016

[B27] Thomson MC Connor SJ D'Alessandro U Rowlingson B Diggle P Cresswell M Greenwood B Predicting malaria infection in Gambian children from satellite data and bed net use surveys: the importance of spatial correlation in the interpretation of results Am J Trop Med Hyg 1999 61 2 8 10432046 10.4269/ajtmh.1999.61.2

[B28] Raso G Silué KD Vounatsou P Singer BH Yapi A Tanner M Utzinger J N'Goran EK Spatial risk profiling of Plasmodium falciparum parasitaemia in a high endemicity area in Côte d'Ivoire Malar J 2009 8 252 10.1186/1475-2875-8-252 19906295 PMC2783037

[B29] Loha E Lindtjørn B Model variations in predicting incidence of Plasmodium falciparum malaria using 1998-2007 morbidity and meteorological data from south Ethiopia Malar J 2010 9 166 10.1186/1475-2875-9-166 20553590 PMC2898788

[B30] Kleinschmidt I Bagayoko M Clarke GP Craig M Le Sueur D A spatial statistical approach to malaria mapping Int J Epidemiol 2000 29 355 361 10.1093/ije/29.2.355 10817136

[B31] Olson SH Gangnon R Elguero E Durieux L Guégan JF Foley JA Patz JA Links between climate, malaria, and wetlands in the Amazon Basin Emerging Infect Dis 2009 15 659 662 10.3201/eid1504.080822 19331766 PMC2671454

[B32] Hay SI Snow RW Rogers DJ Predicting malaria seasons in Kenya using multitemporal meteorological satellite sensor data Trans R Soc Trop Med Hyg 1998 92 12 20 10.1016/S0035-9203(98)90936-1 9692138

[B33] Hay SI Lennon JJ Deriving meteorological variables across Africa for the study and control of vector-borne disease: a comparison of remote sensing and spatial interpolation of climate Trop Med Int Health 1999 4 58 71 10.1046/j.1365-3156.1999.00355.x 10203175 PMC3272404

[B34] Adimi F Soebiyanto RP Safi N Kiang R Towards malaria risk prediction in Afghanistan using remote sensing Malar J 2010 9 125 10.1186/1475-2875-9-125 20465824 PMC2878304

[B35] Gaudart J Touré O Dessay N Dicko AL Ranque S Forest L Demongeot J Doumbo OK Modelling malaria incidence with environmental dependency in a locality of Sudanese savannah area, Mali Malar J 2009 8 61 10.1186/1475-2875-8-61 19361335 PMC2686729

[B36] Peterson AT Ecological niche modelling and understanding the geography of disease transmission Vet Ital 2007 43 393 400 20422515

[B37] Peterson AT Ecologic niche modeling and spatial patterns of disease transmission Emerging Infect Dis 2006 12 1822 1826 10.3201/eid1212.060373 17326931 PMC3291346

[B38] Romaña CA Eco-épidémiologie In Dictionnaire de la pensée médicale 2004 Presses Universitaires de France. Paris: Lecourt 378 382

[B39] Lopes P Lourenço P Sousa C Novo T Rodrigues J Almeida APG Seixas J Modelling patterns of mosquito density based on remote sensing images 2005 In Estoril Congress Center

[B40] Dister SW Fish D Bros SM Frank DH Wood BL Landscape characterization of peridomestic risk for Lyme disease using satellite imagery Am J Trop Med Hyg 1997 57 687 692 9430528 10.4269/ajtmh.1997.57.687

[B41] Frank DH Fish D Moy FH Landscape features associated with lyme disease risk in a suburban residential environment Landscape Ecol 1998 13 27 36 10.1023/A:1007965600166

[B42] Cohen JM Ernst KC Lindblade KA Vulule JM John CC Wilson ML Local topographic wetness indices predict household malaria risk better than land-use and land-cover in the western Kenya highlands Malar J 2010 9 328 10.1186/1475-2875-9-328 21080943 PMC2993734

[B43] Tsayem-Demaze M Manusset S L'agriculture itinérante sur brûlis en Guyane française: la fin des durabilités écologique et socioculturelle? Cah Outre Mer 2008 61 31 48

[B44] Carme B Lecat J Lefebvre P Malaria in an outbreak zone in Oyapock (French Guiana): incidence of malaria attacks in the American Indian population of Camopi. (In French) Med Trop 2005 65 149 154 16038355

[B45] Ehrman FC Ellis JM Young MD Plasmodium vivax Chesson strain Science 1945 101 377 17780321 10.1126/science.101.2624.377

[B46] Hanf M Stefani A Basurko C Nacher M Carme B Determination of the Plasmodium vivax relapse pattern in Camopi, French Guiana Malar J 2009 8 278 278 10.1186/1475-2875-8-278 19961585 PMC2793261

[B47] Stefani A Hanf M Nacher M Girod R Carme B Environmental, entomological, socioeconomic and behavioural risk factors for malaria attacks in Amerindian children of Camopi, French Guiana Malar J 2011 10 246 10.1186/1475-2875-10-246 21861885 PMC3196925

[B48] Jaeger JAG Landscape division, splitting index, and effective mesh size: new measures of landscape fragmentation Landscape Ecol 2000 15 115 130 10.1023/A:1008129329289

[B49] Dray S Legendre P Peres-Neto PR Spatial modelling: a comprehensive framework for principal coordinate analysis of neighbour matrices (PCNM) Ecol Modell 2006 196 483 493 10.1016/j.ecolmodel.2006.02.015

[B50] Wagner HH Spatial covariance in plant communities: integrating ordination, geostatistics, and variance testing Ecology 2003 84 1045 1057 10.1890/0012-9658(2003)084[1045:SCIPCI]2.0.CO;2

[B51] Roux E Venâncio AF Girres J Romaña CA Spatial patterns and eco-epidemiological systems - Part I: Multi-scale spatial modelling of the occurrence of Chagas disease insect vectors Geospatial Health 2011 6 41 51 22109862 10.4081/gh.2011.156

[B52] Moilanen A Nieminen M Simple connectivity measures in spatial ecology Ecology 2002 83 1131 1145 10.1890/0012-9658(2002)083[1131:SCMISE]2.0.CO;2

[B53] Ernst KC Lindblade KA Koech D Sumba PO Kuwuor DO John CC Wilson ML Environmental, socio-demographic and behavioural determinants of malaria risk in the western Kenyan highlands: a case-control study Trop Med Int Health 2009 14 1258 1265 10.1111/j.1365-3156.2009.02370.x 19772547 PMC3109621

[B54] Rozendaal JA Biting and resting behavior of Anopheles darlingi in the Suriname rainforest J Am Mosq Control Assoc 1989 5 351 358 2584968

[B55] Charlwood JD Biological variation in Anopheles darlingi Root Mem Inst Oswaldo Cruz 1996 91 391 398 9070397 10.1590/s0074-02761996000400001

[B56] Tadei WP Thatcher BD Santos JM Scarpassa VM Rodrigues IB Rafael MS Ecologic observations on anopheline vectors of malaria in the Brazilian Amazon Am J Trop Med Hyg 1998 59 325 335 9715956 10.4269/ajtmh.1998.59.325

[B57] Tadei WP Dutary Thatcher B Malaria vectors in the Brazilian amazon: Anopheles of the subgenus Nyssorhynchus Rev Inst Med Trop Sao Paulo 2000 42 87 94 10.1590/S0036-46652000000200005 10810323

[B58] Olson SH Gangnon R Silveira GA Patz JA Deforestation and malaria in Mâncio Lima County, Brazil Emerging Infect Dis 2010 16 1108 1115 20587182 10.3201/eid1607.091785 PMC3321904

[B59] Vittor AY Gilman RH Tielsch J Glass G Shields T Lozano WS Pinedo-Cancino V Patz JA The effect of deforestation on the human-biting rate of Anopheles darlingi, the primary vector of Falciparum malaria in the Peruvian Amazon Am J Trop Med Hyg 2006 74 3 11 16407338

[B60] Vittor AY Pan W Gilman RH Tielsch J Glass G Shields T Sánchez-Lozano W Pinedo VV Salas-Cobos E Flores S Patz JA Linking deforestation to malaria in the Amazon: characterization of the breeding habitat of the principal malaria vector, Anopheles darlingi Am J Trop Med Hyg 2009 81 5 12 19556558 PMC3757555

[B61] Patz JA Graczyk TK Geller N Vittor AY Effects of environmental change on emerging parasitic diseases Int J Parasitol 2000 30 1395 1405 10.1016/S0020-7519(00)00141-7 11113264

[B62] Raccurt CP [Malaria, anopheles, the anti-malaria campaign in French Guyana: between dogmatism and judgment] Med Trop 1997 57 401 406 9612784

[B63] Hiwat H Issaly J Gaborit P Somai A Samjhawan A Sardjoe P Soekhoe T Girod R Behavioral heterogeneity of Anopheles darlingi (Diptera: Culicidae) and malaria transmission dynamics along the Maroni River, Suriname, French Guiana Trans R Soc Trop Med Hyg 2010 104 207 213 10.1016/j.trstmh.2009.07.007 19732925

[B64] Fouque F Gaborit P Carinci R Issaly J Girod R Annual variations in the number of malaria cases related to two different patterns of Anopheles darlingi transmission potential in the Maroni area of French Guiana Malar J 2010 9 80 10.1186/1475-2875-9-80 20307300 PMC2859772

